# Air pollution and respiratory outcomes in COPD above and below current environmental protection agency standards

**DOI:** 10.1097/EE9.0000000000000491

**Published:** 2026-06-01

**Authors:** Laura C. Myers, Sahithi Chimmula, David Schlessinger, Kathleen A. Daly, Crystal A. Hsiao, Joel Schwartz, Stephen K. Van Den Eeden, Stephen Sidney, Kamala Deosaransingh, Stacey Alexeeff

**Affiliations:** a The Division of Research, Kaiser Permanente Northern California, Pleasanton, California; b Department of Environmental Health, Harvard Chan School of Public Health, Boston, Massachusetts

**Keywords:** COPD, Particulate matter air pollution, Respiratory outcomes

## Abstract

**Background:**

Rigorous studies are needed to defend the current National Ambient Air Quality Standards and prompt policymakers to acknowledge a dose-response relationship between air pollution and respiratory outcomes. In the most recent Environmental Protection Agency comprehensive review, scientists determined there was insufficient evidence to claim a causal relationship between chronic particulate matter air pollution (PM_2.5_) and respiratory outcomes, despite acknowledging one for cardiovascular outcomes (Integrated Science Assessment, *Table 5–27*).

**Methods:**

This study examined the associations between chronic PM_2.5_ levels and respiratory outcomes in 169,713 patients within Kaiser Permanente Northern California who had chronic obstructive pulmonary disease (COPD) between 2007 and 2016.

**Results:**

For every 10 μg/m^3^ rise in average yearly PM_2.5_, there was a statistically significant increase in relative risk of death (hazard ratio [HR] = 1.15; 95% confidence interval [CI] = 1.10, 1.20), respiratory-related death (HR = 1.22; 95% CI = 1.15, 1.30), COPD exacerbation (HR = 1.35; 95% CI = 1.29, 1.41), and the combined endpoint of COPD exacerbation or respiratory-related hospitalization with any-cause death as competing risk (HR = 1.30; 95% CI = 1.25, 1.35). We demonstrate a dose-response relationship between 1-year average PM_2.5_ exposure and respiratory outcomes for key regulatory categories of PM_2.5_.

**Conclusions:**

These findings add rigorous evidence to support the continuation of the current annual PM_2.5_ standard, which was lowered to 9 μg/m^3^ in 2024. Our *novel contribution* is documenting the dose-response relationship between chronic PM_2.5_ and multiple respiratory outcomes in a longitudinal cohort with patients followed for up to 10 years.

**What this study adds**

These findings add rigorous evidence to support the continuation of the current annual PM_2.5_ standard, which was lowered to 9 μg/m^3^ in 2024. Given the increased risk of respiratory outcomes associated with low PM_2.5_ (8.1–9 μg/m^3^) compared to very low, these findings might even support a *further lowering* of the annual standard to 8 μg/m^3^ when combined with existing literature. Our *novel contribution* is documenting the dose-response relationship between chronic PM_2.5_ and multiple respiratory outcomes in a longitudinal cohort with patients followed for up to 10 years, which provides evidence for causality during the next scientific review of the National Ambient Air Quality Standards.

## Introduction

Ample studies have described the association between exposure to particulate matter air pollution with diameter ≤2.5 microns (PM_2.5_) and respiratory outcomes.^1–3^ However, in the most recent Environmental Protection Agency comprehensive review, scientists determined there was insufficient evidence to claim a causal relationship between chronic PM_2.5_ and respiratory outcomes, despite acknowledging one for cardiovascular outcomes (Integrated Science Assessment, *Table 5-27*).^4^ Rigorous studies are needed to defend the current National Ambient Air Quality Standards (1-year average PM_2.5_ ≤9 μg/m^3^),^5^ which were lowered in 2024, through inevitable changes in federal administrations.^5^ Additionally, analyses specifically assessing causality are needed. We leveraged a longitudinal cohort of patients with chronic obstructive pulmonary disease (COPD) to assess the dose-response relationship between PM_2.5_ and various respiratory outcomes, which is a retrospective analysis that can be performed to support the notion of causality.^6^

## Methods

We performed retrospective analyses of existing data (IRB1339692-6 with a waiver of informed consent).

The study population included adult members of Kaiser Permanente Northern California aged ≥18 years old with COPD between 2007 and 2016 and lived in Northern California for ≥1 year. COPD was previously defined by the following diagnosis codes (ICD-9 491, 492, 496; ICD-10 J41, J43, J44). Patients entered the cohort on the day they met the above criteria. Follow-up continued until the end of membership, relocation out of the study region, death or December 31, 2016, whichever was earliest.

The exposure was individual-level, time-varying, 1-year average PM_2.5_ exposure based on patients’ home address. PM_2.5_ levels were previously obtained from an ensemble model with 1 × 1 km resolution that combines satellite-based aerosol optical depth measurements, absorbing aerosol index data, satellite-based surface reflectance data, chemical transport model outputs, meteorologic data and land use data (performance R^2^ = 0.89 for 1-year PM_2.5_ predictions using 10-fold cross validation). Exposure was updated monthly and accounted for patients moving.

We evaluated the following outcomes:

1) any-cause death.
2) respiratory-related death^7^: death associated with lung cancer (C33, C34.0–C.34.9), COPD (J42, J43.9, J44.0–J44.9, J47), asthma (J45.0, J45.9, J46), pneumonia (J10.1, J11.0–J11.1, J13–J14, J15.2–J15.9, J18.0–J18.9), interstitial disease (J84.0, J84.1, J84.9) and other respiratory disease (J00–J06, J20–J39, J60–J99).
3) COPD exacerbation^8^: acute care visit with COPD diagnosis in primary position, acute care visit with acute respiratory failure in primary position and COPD diagnosis code in any position, acute care visit with acute respiratory failure in primary position with acute care visit for COPD within 7 days, or outpatient visit with COPD diagnosis code and pharmacy dispense record for oral corticosteroid or a COPD-guideline concordant antibiotic.
4) respiratory-related hospitalization: hospitalizations with the following respiratory-related diagnosis codes (J00–J99).

We fit Cox proportional hazards models that sequentially adjusted for more covariates (Model 1: age, sex, race/ethnicity, and calendar year, Model 2: additionally adjusted for smoking, body mass index, baseline comorbidities, previous need for supplemental oxygen, Model 3: additionally adjusted for neighborhood education, Medicaid insurance). We accounted for age based on time lapsed in the study. *The primary analysis was predefined as Model 3 for the outcome of COPD exacerbation with death as a competing risk.* We estimated continuous linear associations and categorical associations; we tested for linearity using restricted cubic splines. The cutoffs for categorical analysis were based on the past and current regulatory standards from the Environmental Protection Agency to be policy relevant (very low 1-year average PM_2.5_ ≤8 μg/m^3^, low 8.1–9 μg/m^3^, moderate 9.1–12.0μg/m^3^, high >12 μg/m^3^). Alpha of 0.05 and SAS software (Version 9.4, Cary, NC) were used.

We evaluated the dose-response relationship, which is one way retrospectively to support the notion of causality in the absence of randomized clinical trials.^6^

## Results

**Figure 1.**
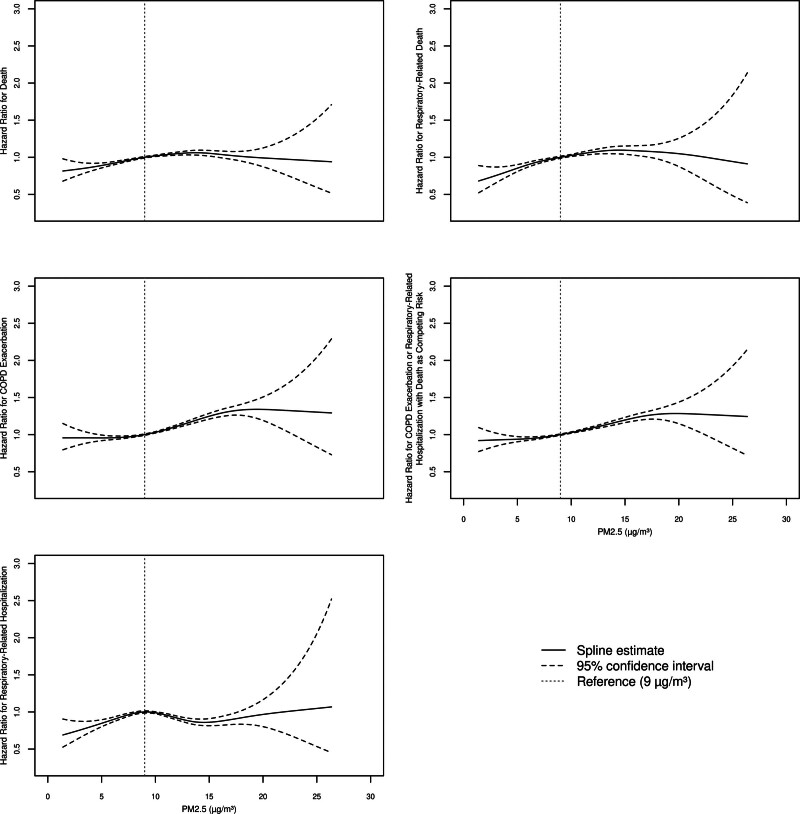
**Linearity of the relationship between chronic PM_2.5_ and outcomes using restricted cubic splines.** The *P*-value for linearity for each outcome was death (*P* = 0.000000002), respiratory-related death (*P* = 0000000006), COPD exacerbation (*P* < 0.000000002), COPD exacerbation or respiratory-related hospitalization with death as competing risk (*P* < 0.000000002), respiratory-related hospitalization (*P* = 0.46).

**Figure 2.**
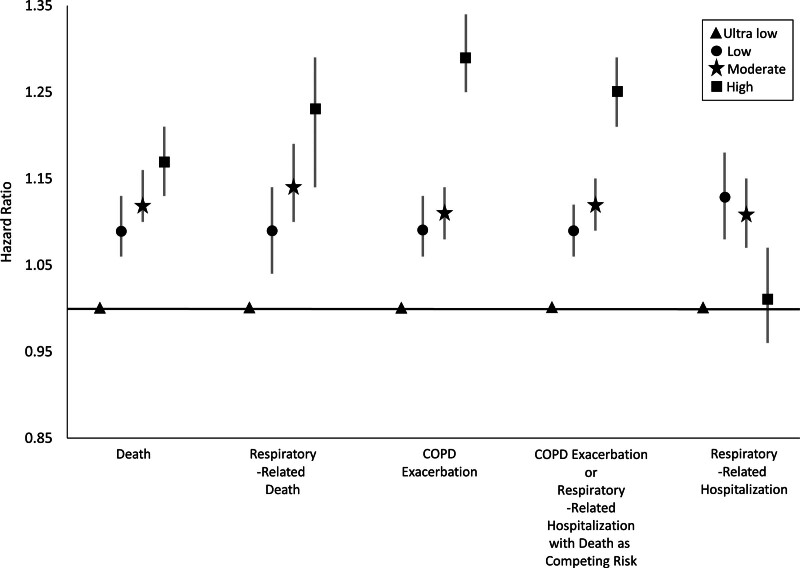
**Dose-response relationship between chronic PM_2.5_ and various respiratory outcomes.** The hazard ratios and 95% confidence intervals are shown and grouped by respiratory outcome. The reference group is very low 1-year average PM_2.5_ (≤8 μg/m^3^). Circles represent low 1-year average PM_2.5_ (8.1–9 μg/m^3^). Stars represent a moderate 1-year average PM_2.5_ (9.1–12.0 μg/m^3^). Squares represent high 1-year average PM_2.5_ (>12 μg/m^3^).

The cohort consisted of 169,713 patients (mean age: 60.8 years [SD: 15.6], 62.4% were current or former smokers). Full baseline characteristics were previously described in a study examining cardiovascular outcomes.^9^ In terms of baseline 1-year average PM_2.5_, there were 13,665 (8%) in the very low, 18,069 (11%) in the low, 107,845 (63%) in the moderate and 30,134 (18%) in the high group; the distribution had a median of 10.2 μg/m^3^ (interquartile range: 9.31, 11.4). For each increase of 10 μg/m^3^ in average yearly PM_2.5_ in fully adjusted models_,_ there was a statistically significant increase in relative risk of death (hazard ratio [HR] = 1.15; 95% confidence interval [CI] = 1.10, 1.20), respiratory-related death (HR = 1.22; 95% CI = 1.15, 1.30), COPD exacerbation (HR = 1.35; 95% CI = 1.29, 1.41), and the combined endpoint of COPD exacerbation or respiratory-related hospitalization with any-cause death as competing risk (HR = 1.30; 95% CI = 1.25, 1.35) but not respiratory-related hospitalization alone (HR = 0.98; 95% CI = 0.91, 1.04) (**Table 1**). The association between 1-year average PM_2.5_ and respiratory-related hospitalizations was nonlinear (*P* for linearity 0.46, **Figure 1**). **Figure 2** shows the dose response relationship between 1-year average PM_2.5_ exposure and respiratory outcomes for key regulatory categories.

**Table 1.** Associations between chronic PM_2.5_ and various respiratory outcomes

Outcomes	Numbers of events	Model 1^a^ HR (95% CI)	Model 2^b^ HR (95% CI)	Model 3^c^ HR (95% CI)
Death	40,392	1.23 (1.17, 1.28)	1.23 (1.18, 1.28)	1.15 (1.10, 1.20)
Respiratory-related death	19,738	1.32 (1.25, 1.41)	1.33 (1.25, 1.41)	1.22 (1.15, 1.30)
COPD exacerbation	46,019	1.45 (1.39, 1.49)	1.43 (1.37, 1.49)	1.35 (1.29, 1.41)
COPD exacerbation or respiratory-related hospitalization with death as competing risk	51,162	1.40 (1.34, 1.45)	1.37 (1.32, 1.43)	1.30 (1.25, 1.35)
Respiratory-related hospitalization	19,087	1.08 (1.10, 1.15)	1.06 (1.00, 1.13)	0.98 (0.91, 1.04)

a Model 1: Adjusted for age, sex, race/ethnicity, calendar year.

b Model 2: Adjusted for age, sex, race/ethnicity, calendar year, smoking, body mass index, supplemental oxygenation use, baseline COPS2 (index of comorbidity burden).

c Model 3: Adjusted for age, sex, race/ethnicity, calendar year, smoking, body mass index, supplemental oxygenation use, baseline COPS2 (index of comorbidity burden), neighborhood education, and Medicaid insurance.

CI indicates confidence interval; COPD, chronic obstructive pulmonary disease; HR, hazard ratio.

## Discussion

We provide evidence of a dose-response relationship between 1-year average PM_2.5_ and several respiratory outcomes, including COPD exacerbation. The results were generally consistent across multiple respiratory outcomes. The nonsignificant relationship and lack of dose response for respiratory-related hospitalization may be explained by the nonlinearity detected in the cubic spline analysis.

These findings add rigorous evidence to support the continuation of the current annual PM_2.5_ standard, which was lowered to 9 μg/m^3^ in 2024. Given the increased risk of respiratory outcomes associated with low PM_2.5_ (8.1–9 μg/m^3^) compared with very low, these findings might even support a *further lowering* of the annual standard to 8 μg/m^3^ when combined with existing literature.^1–3^ Our *novel contribution* is documenting the dose-response relationship between chronic PM_2.5_ and multiple respiratory outcomes in a longitudinal cohort with patients followed for up to 10 years.

There are several strengths and limitations. We leveraged highly resolved exposure data and unique, diverse longitudinal patients in order to answer a pressing environmental public health question. In so doing, we were able to observe 1 million person-years with an average follow-up time of 6.5 years/person. We specifically utilized an existing cohort from 2007 to 2016 in order to study the dose-response relationship with a high exposure group (>12 μg/m^3^), which requires historical data. Adult Kaiser Permanente members have been shown to reflect the demographic characteristics of the Northern California population.^10^ Limitations include (1) lack of granularity into how much time patients spend indoors and near their home, (2) unmeasured confounding, and (3) lack of spirometry data. However, patients with COPD have been shown to spend ≥65% of time at home,^11^ we adjusted for key confounders (smoking status, oxygen dependence, comorbidity burden), and we had previously done a chart review validation of the diagnosis code-based cohort derivation algorithm where we assessed airflow obstruction by spirometry.^9^ Finally, while the dose response results were mostly consistent, a dose response relationship was not seen with the respiratory-related outcome. We feel this is due to the nonlinearity of the relationship, that is, a more complex interplay between exposure and hospitalization exists, but interestingly, the dose response relationship prevails when used as part of a composite outcome. Further investigation into patient behavior and clinician admitting patterns are warranted to further understand this.

In conclusion, we demonstrate a dose-response relationship between chronic PM_2.5_ and multiple respiratory outcomes. This finding strengthens the argument in favor of there being a causal association between chronic PM_2.5_ and respiratory outcomes. We expect that this study will become part of the next Integrated Science Assessment and inform future air pollution policy in the United States.

## Conflicts of interest statement

The authors declare that they have no conflicts of interest with regard to the content of this report.

## References

[1] Ostro B Lipsett M Reynolds P. Long-term exposure to constituents of fine particulate air pollution and mortality: results from the California Teachers Study. Environ Health Perspect. 2010;118:363–369. 20064787 10.1289/ehp.0901181 PMC2854764

[2] Hart JE Garshick E Dockery DW Smith TJ Ryan L Laden F. Long-term ambient multipollutant exposures and mortality. Am J Respir Crit Care Med. 2011;183:73–78. 20656944 10.1164/rccm.200912-1903OC PMC3040395

[3] Pinault L Tjepkema M Crouse DL. Risk estimates of mortality attributed to low concentrations of ambient fine particulate matter in the Canadian community health survey cohort. Environ Health. 2016;15:18. 26864652 10.1186/s12940-016-0111-6 PMC4750218

[4] Environmental Protection Agency. Integrated Science Assessment for Particulate Matter. 2019. 36630543

[5] Sin DD Doiron D Agusti A; GOLD Scientific Committee. Air pollution and COPD: GOLD 2023 committee report. Eur Respir J. 2023;61:2202469. 36958741 10.1183/13993003.02469-2022

[6] Hernán MA. Methods of public health research–strengthening causal inference from observational data. N Engl J Med. 2021;385:1345–1348. 34596980 10.1056/NEJMp2113319

[7] Stavem K Johannessen A Nielsen R Gulsvik A. Respiratory symptoms and respiratory deaths: a multi-cohort study with 45 years observation time. PLoS One. 2021;16:e0260416. 34807953 10.1371/journal.pone.0260416 PMC8608323

[8] Palli SR Zhou S Shaikh A Willey VJ. Effect of compliance with GOLD treatment recommendations on COPD health care resource utilization, cost, and exacerbations among patients with COPD on maintenance therapy. J Manag Care Spec Pharm. 2021;27:625–637. 33779246 10.18553/jmcp.2021.20390 PMC10394222

[9] Alexeeff SE Deosaransingh K Liao NS Van Den Eeden SK Schwartz J Sidney S. Particulate matter and cardiovascular risk in adults with chronic obstructive pulmonary disease. Am J Respir Crit Care Med. 2021;204:159–167. 33662228 10.1164/rccm.202007-2901OC PMC8650791

[10] Davis AC Voelkel JL Remmers CL Adams JL McGlynn EA. Comparing Kaiser Permanente members to the general population: implications for generalizability of research. Perm J. 2023;27:87–98. 37170584 10.7812/TPP/22.172 PMC10266863

[11] Spalt EW Curl CL Allen RW. Time-location patterns of a diverse population of older adults: the multi-ethnic study of atherosclerosis and air pollution (MESA Air). J Expo Sci Environ Epidemiol. 2016;26:349–355. 25921083 10.1038/jes.2015.29 PMC4641054

